# Determination of Photoperiod-Sensitive Phase in Chickpea (*Cicer arietinum* L.)

**DOI:** 10.3389/fpls.2016.00478

**Published:** 2016-04-11

**Authors:** Ketema Daba, Thomas D. Warkentin, Rosalind Bueckert, Christopher D. Todd, Bunyamin Tar’an

**Affiliations:** ^1^Crop Development Centre/Department of Plant Sciences, University of SaskatchewanSaskatoon, SK, Canada; ^2^Department of Biology, University of SaskatchewanSaskatoon, SK, Canada

**Keywords:** adaptation, flowering, photoperiod-insensitive phase, long days, short days

## Abstract

Photoperiod is one of the major environmental factors determining time to flower initiation and first flower appearance in plants. In chickpea, photoperiod sensitivity, expressed as delayed to flower under short days (SD) as compared to long days (LD), may change with the growth stage of the crop. Photoperiod-sensitive and -insensitive phases were identified by experiments in which individual plants were reciprocally transferred in a time series from LD to SD and vice versa in growth chambers. Eight chickpea accessions with differing degrees of photoperiod sensitivity were grown in two separate chambers, one of which was adjusted to LD (16 h light/8 h dark) and the other adjusted to SD (10 h light/14 h dark), with temperatures of 22/16°C (12 h light/12 h dark) in both chambers. The accessions included day-neutral (ICCV 96029 and FLIP 98-142C), intermediate (ICC 15294, ICC 8621, ILC 1687, and ICC 8855), and photoperiod-sensitive (CDC Corinne and CDC Frontier) responses. Control plants were grown continuously under the respective photoperiods. Reciprocal transfers of plants between the SD and LD photoperiod treatments were made at seven time points after sowing, customized for each accession based on previous data. Photoperiod sensitivity was detected in intermediate and photoperiod-sensitive accessions. For the day-neutral accession, ICCV 96029, there was no significant difference in the number of days to flowering of the plants grown under SD and LD as well as subsequent transfers. In photoperiod-sensitive accessions, three different phenological phases were identified: a photoperiod-insensitive pre-inductive phase, a photoperiod-sensitive inductive phase, and a photoperiod-insensitive post-inductive phase. The photoperiod-sensitive phase extends after flower initiation to full flower development. Results from this research will help to develop cultivars with shorter pre-inductive photoperiod-insensitive and photoperiod-sensitive phases to fit to regions with short growing seasons.

## Introduction

In western Canada, the short crop growing season available for chickpea (110–120 days) often coincides with end-of-season frost resulting in severe losses in grain yield and quality (Warkentin et al., 2003). In order to maximize crop yield through agronomic management or plant breeding, the phenology of the crop must be well matched to the resources and constraints of the production environment (Summerfield et al., 1996; Levy and Dean, 1998; Warkentin et al., 2003). All flowering plants undergo several developmental transitions during their life cycle which can be divided into three major physiological developmental phases: a vegetative phase, from emergence to flower initiation, the reproductive phase, from floral initiation to anthesis, and physiological maturity, from anthesis to seed filling (Ritchie, 1991; Ritchie et al., 1998). The vegetative growth phase is comprised of the basic vegetative phase and the photoperiod-sensitive phase (Vergara and Chang, 1985).

The transition from the vegetative to reproductive phase is a major developmental switch in the plant’s life cycle (Levy and Dean, 1998). This transition is crucial for survival because plants normally time the onset of flowering to suitable environmental conditions. Many plant species have evolved the ability to initiate flowering in response to environmental factors such as changes in photoperiod and temperature. The beginning stage of flowering commands the start of the seed-set period, and, thus, is a key stage in yield formation (Lejeune-Hénaut et al., 1999; Putterill et al., 2004). Flower development and the seed-set stages are greatly impeded by stress such as drought and frost, so flowering and seed development must be completed during favorable growing conditions. Timely flowering and maturity in relation to the available growing season in a particular location are essential for high yield potential from annual crops (Bunting, 1975). Understanding the photoperiod-sensitive phase of a photoperiodic plant would allow better crop management strategy to either promote early flowering to reduce crop duration time, or to intentionally delay flowering (Warner, 2009).

In experiments on rice (*Oryza sativa* L.), where transfers were made between long days (LD) and short days (SD) and vice versa, the photoperiod-sensitive phase was flanked by two photoperiod-insensitive phases (Yin, 2008). In wheat (*Triticum aestivum* L.), a LD plant, the full pre-anthesis period was found to be divided into three sub-phases: from sowing to the terminal spikelet, from terminal spikelet initiation to heading, and from heading to anthesis indicating stage-dependence of plant responsiveness to temperature (Slafer and Rawson, 1995). In this crop, the flowering response was affected by temperature throughout their life cycles (Slafer and Rawson, 1994). In SD crop species such as cowpea (*Vigna unguiculata* L. Walp.) and soybean (*Glycine max* L. Merr.), there was a temperature-dependent critical photoperiod phase. Beyond the critical point, time to flowering was solely a function of mean temperature (Hadley et al., 1984). Maize (*Zea mays* L.) is also sensitive to photoperiod during tassel initiation (Kiniry et al., 1983). In rice and soybean, the photoperiod influence even extends for some time beyond the phase of floral initiation (Collinson et al., 1992; Ellis et al., 1992).

Earlier studies on wheat reported that exposure to long photoperiod significantly reduced the time to heading (Slafer and Rawson, 1994, 1997). Estimation of phasic development is crucial for accurate modeling of plant development and yield components, as well as for evaluating cultivar adaptation and scheduling cultural practices (Shaykewich, 1995). Quantitative models to determine the developmental phases in different plants were developed using different parameters and plant materials. Flower development phases were quantified using four parameters; a_1_ (the photoperiod-insensitive pre-inductive phase), *I*_s_ (the photoperiod-sensitive inductive phase in LD and SD), and a_3_ the photoperiod-insensitive post-inductive phases in LD and SD (Ellis et al., 1992). Similarly, photoperiod-sensitive inductive phases in LD and SD were denoted as *I*_2L_ and *I*_2S_, respectively, by following the procedure developed by Yin (2008).

Short-day does not delay flowering in a LD plant if exposure is restricted to the photoperiod-insensitive pre-inductive phase or the photoperiod-insensitive phase of flower development. However, time to flower is delayed if the plant is exposed to SD during the photoperiod-sensitive phase. Similarly, LD will only hasten flowering in LD plants if the plants are exposed to the photoperiod when they are at the photoperiod-sensitive stage as summarized by Adams et al. (2001, 2003). The duration of the photoperiod-sensitive phases can be determined by examining data on the time to first flower opening of plants transferred between SD and LD at different times (Wang et al., 1997; Yin et al., 1997; Adams et al., 2001). Chickpea is inherently considered as a LD plant (Soltani et al., 2004). Chickpea accessions with day neutral, intermediate, and highly sensitive response to photoperiod were recently reported by Daba et al. (2015). Little is known about the duration of the photoperiod-sensitive and -insensitive phases in chickpea. Therefore, the reciprocal transfer technique was used to quantify and identify the timing and duration of the photoperiod-sensitive phase and the time of floral initiation in chickpea. The objectives of this research were to determine the timing and duration of the photoperiod-sensitive and photoperiod-insensitive phase in selected chickpea accessions representative of different maturity classes, and to establish whether photoperiod sensitivity ends at floral initiation or extends into the phase of flower development.

## Materials and Methods

### Accessions Evaluated

Eight diverse chickpea accessions namely: ICCV 96029 (S1), FLIP 98-142C (S2), ICC 15294 (I1), ICC 8621 (I2), ILC 1687 (I3), and ICC 8855 (I4), CDC Corinne (S1), and CDC Frontier (S2) collected from the gene banks of the International Crops Research Institute for the Semi-Arid Tropics (ICRISAT), India and the International Center for Agricultural Research in the Dry Areas (ICARDA), together with cultivars developed at the Crop Development Centre, University of Saskatchewan were used in this research (**Table 1**). These eight genotypes are referred to as ‘accessions’ throughout this paper.

**Table 1:** Market class, origin, and potential photoperiod sensitivity group of chickpea accessions used in the determination of photoperiod sensitivity phase by reciprocally transferring plants from LD to SD.

Accessions	Market class	Origin	Difference in number of days to flowering (SD–LD)	Photoperiod sensitivity group
CDC Corinne	Desi	CDC, University of Saskatchewan	62	S1
CDC Frontier	Kabuli	CDC, University of Saskatchewan	60	S2
ICC 15294	Desi	Iran	42	I1
ICC 8621	Desi	Ethiopia	20	I2
ILC 1687	Kabuli	Afghanistan	27	I3
ICC 8855	Kabuli	Afghanistan	40	I4
ICCV 96029	Desi	ICRISAT	2	N1
FLIP 98-142C	Kabuli	ICARDA	10	N2

The accessions were grown in two separate growth chambers: one of the chambers was adjusted to SD of 10 h light period (SD) and the other chamber was adjusted to LD of 16 h light period (LD). The chambers were maintained at day/night temperatures of 22/16°C (12/12 h). The 12/12 h cycle was used to avoid confounding effects of asynchrony between thermal and photoperiod factors (Roberts et al., 1985; Yin, 2008). Both growth chambers were equipped with inflorescent light bulbs with total light irradiance of 370 μmol m^-2^s^-1^ at just above the plant canopy.

Three seeds of each accession were planted in 3.8 L pots containing Sunshine mix #4 (Sun Gro, Seba Beach, AB, Canada). Seedlings were thinned to two plants per pot 2 weeks after sowing or after full emergence of the seedlings. Starting from 1 week after crop emergence, the plants were watered every 2–3 days based on the growth stage and water use of each accession. Once a week a quick release fertilizer (20 N:20 P_2_O_5_:20 K_2_O) prepared at a concentration of 3 g L^-1^ was applied at a rate of 100 ml per pot starting 1 week after emergence.

**FIGURE 1:**
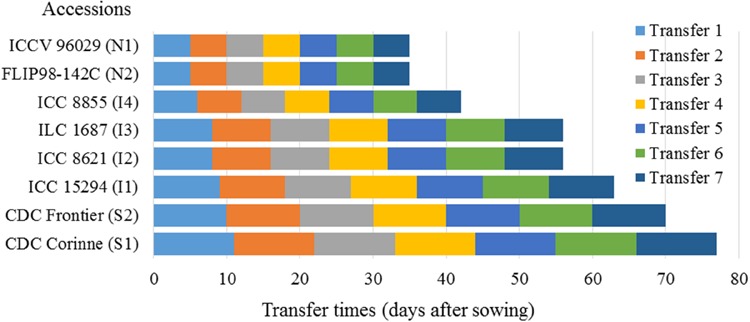
**Chickpea accessions used in the determination of photoperiod sensitivity phase and days from sowing to transfer for plants moved from LD to SD and vice versa.** Control plants of each accession were continuously grown under LD and SD.

A total of three pots with two plants per pot were assigned for each treatment. Each transfer treatment, therefore, contained a total of six plants. A total of six pots were also assigned as a control for each accession. Within the growth chambers, a total of 27 pots, for each of the seven transfers and the control pots for each accession were completely randomized. Control plants were continuously grown at LD and SD (**Table 2** and **Figure 1**). Transfer times for these accessions were customized based on their differences in the number of days to flowering under short compared to LD (Daba et al., 2015). Once plants had been transferred, they were continuously maintained in the new chamber under either LD or SD. The entire experiment was carried out in two runs (two time replicates).

**Table 2:** Chickpea accessions used in the determination of photoperiod-sensitivity phase and days from sowing to transfer (tc) for plants moved from LD to SD and vice versa.

Accessions	Transfer times (days after sowing)							
CDC Corinne (S1)	0	11	22	33	44	55	66	77
CDC Frontier (S2)	0	10	20	30	40	50	60	70
ICC 15294 (I1)	0	9	18	27	36	45	54	63
ICC 8621 (I2)	0	8	16	24	32	40	48	56
ILC 1687 (I3)	0	8	16	24	32	40	48	56
ICC 8855 (I4)	0	6	12	18	24	30	36	42
ICCV 96029 (N1)	0	5	10	15	20	25	30	35
FLIP 98-142C (N2)	0	5	10	15	20	25	30	35

*0 day to transfer refers to control plants which were grown under SD or LD throughout.*

### Data Collection and Analysis

#### Flower Bud Initiation and Full Flower Opening

First flower bud initiation stage and full open flower appearance (corolla visible) were recorded for each accession. Samples of flower buds were carefully collected from each control and transferred plants in both photoperiod treatments. The stipules were dissected using blades to expose the shoot apex and newly initiated phytomers and a node subtending a leaf primordium, and an auxiliary vegetative or reproductive bud and were evaluated under the microscope. In cases of the death of the first initiated buds, the subsequently formed buds were dissected. Upon seeing fully developed anthers bound by a fully developed calyx, the flower bud initiation stages (as the number of days to flower bud initiation from days of seeding) were declared. Days to first flowering for the transfer and control plants in LD and SD were recorded when a fully opened flower appeared on each plant. Days to flower bud initiation and first flowering of the control plants in short and LD photoperiods were used for comparison.

#### Identification of Slope Coefficients, *y*-Intercepts and Hinges

**FIGURE 2:**
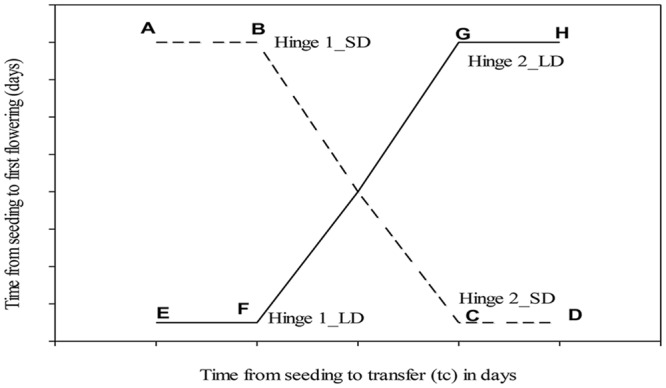
**Schematic representation of the hypothetical response of the time from sowing to first flowering for plants transferred from a SD to a LD (a solid line) and from a LD to a SD regime (a dotted line) at various time intervals from seeding to first flowering.** The first and second hinges for LD (Hinge 1_LD and Hinge 2_LD) were identified at the hinge point at the intersection of line segments of EF and GF. Similarly, the first and second hinges for SD (Hinge 1_SD and Hinge 2_SD) were identified as a hinge point at the intersection of lines AB and CB. The duration of the photoperiod-sensitive inductive phase (durations *I*_2s_ and *I*_2L_ in short and LD, respectively) was included between a photoperiod-insensitive pre-inductive and a photoperiod-insensitive post-inductive phase. The three sub-phases under the SD conditions are indicated by the linear segments ‘AB,’ ‘BC,’ and ‘CD,’ respectively, and those under the LD conditions are indicated by linear segments ‘EF,’ ‘FG,’ and ‘GH,’ respectively (Ellis et al., 1992; Adams et al., 2001, 2003; Yin, 2008).

Segmented linear regression analysis was conducted for each accession in order to determine the differences in the photoperiod-sensitive and photoperiod-insensitive phases in the chickpea accessions reciprocally transferred from LD to SD. The hypothetical response of the time from sowing to first flowering for plants transferred from a SD to a LD and from a LD to a SD regime at various time intervals from seeding to first flowering were illustrated in **Figure 2**. Control plants continuously grown under SD are indicated by point A, and those grown under LD are indicated by point E. The intersection point of linear segments AB and CB representing the first hinge point for transfer from LD to SD, whereas the intersection point between linear segments of EF and FG represented the first hinge for transfers from SD to LD. Accordingly, the first hinge was calculated as a function of days of transfer from seeding to days to flowering from seeding where the increase or decrease in the slope between the linear segments occurred.

**FIGURE 3:**
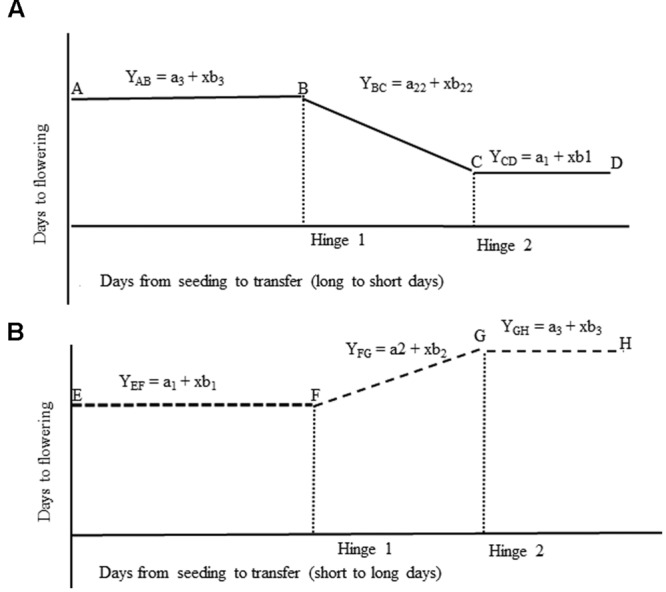
**Diagrammatic representation for linear regression equations used in the determination of hinge 1 and hinge 2 in accessions transferred from (A) LD to SD, and (B) from SD to LD.** For plants transferred from LD to SD **(A)**. AB – The photoperiod-insensitive pre-inductive phase, BC – The photoperiod-sensitive inductive phase, CD – The photoperiod-insensitive post-inductive phase. For plants transferred from SD to LD **(B)**. EF – The photoperiod-insensitive pre-inductive phase, FG – The photoperiod-sensitive inductive phase, GH – The photoperiod-insensitive post-inductive phase.

The two-phase or piecewise regression (hinged regression) has been described by Breiman (1993) and used by Dunnigan et al. (1997), and Kenesei and Abonyi (2013). The individual linear segments were then used to determine the photoperiod-sensitive and -insensitive phases in chickpea accessions following a procedure described by Wang et al. (1997). The intersection of the two linear equations, the hinge, was positioned to identify the changes in slope coefficients and *y*-intercepts using simultaneous equations for each part of the regression models (**Figure 3**). The models were interactively congregated in PROC MODEL of SAS version 9.3 (SAS Institute Inc., 2009; SAS Institute Inc., Cary, NC, USA). Hinged regression analyses were conducted for each accession to determine the parameters (a1, b1, a2, b2, hinge 1, a22, b22, a3, b3, and hinge 2) where a and b represent intercept and slope coefficients of the respective segments of the modeled line. To test if regression intercept and slope coefficient varied among accessions, analyses of variance were conducted using PROC GLM of SAS version 9.3 (SAS Institute Inc., 2009; SAS Institute Inc., Cary, NC, USA).

#### Identification of Photoperiod-Sensitive and Photoperiod-Insensitive Phases

**FIGURE 4:**
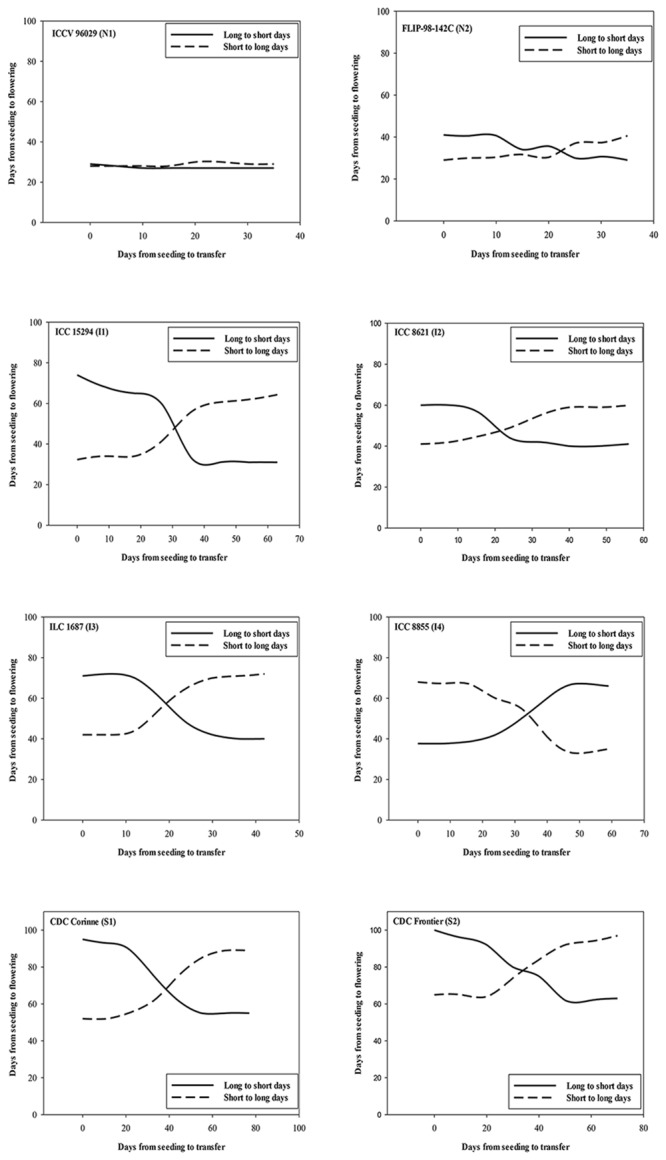
**The effect of transferring the plants at varying intervals from LD to SD (solid line) and SD to LD (dashed line) on the number of days to first flower opening for each of day neutral (ICCV 96029, FLIP 98-142C), intermediate (ICC 15294, ICC 8621, ILC 1687, and ICC 8855) and photoperiod-sensitive (CDC Corinne and CDC Frontier) accessions**.

Data on days to flowering for the transfer and control plants (**Figure 4**) were analyzed using PROC NON-LINEAR of SAS version 9.3 (SAS Institute Inc., 2009; SAS Institute Inc., Cary, NC, USA). Initially, separate data analyses were conducted for each time replicate. There was no significant difference between the results of the two time replicates. Homogeneity of variance for each time replicate was validated using Levene’s Test. Thus, a combined data analysis was conducted using the average data of the replications in both time replicates for each accession transferred from LD to SD and vice versa, and the control plants.

The time to flower in the eight chickpea accessions in the successive photoperiod-transfer and control treatments were modeled against the time to transfer after seeding following the procedure of Yin (2008) as illustrated in **Figure 2**, which combines data from transfers of LD to SD and short to LD in a single curve fitting procedure.

In the analysis, *f*_L_ was assigned as the duration from sowing to flowering for the LD and can be written as: *f*_L_ = *I*_1L_ + *I*_2L_ + *I*_3L_; where: *I*_1L_ is the first sub-phase, a photoperiod-insensitive pre-inductive phase; *I*_2L_ is the second sub-phase, a photoperiod-sensitive inductive phase; *I*_3L_ is the third sub-phase, a photoperiod-insensitive post-inductive phase under LD conditions (Yin, 2008). Similarly *f*_S_ was assigned as the duration from sowing to flowering for the SD and the expression can be written as: *f*_L_ = *I*_1S_ + *I*_2S_ + *I*_3S_; where: *I*_1S_ is the first sub-phase a photoperiod-insensitive pre-inductive phase; *I*_2S_ is the second sub-phase a photoperiod-sensitive phase; *I*_3S_ is the third sub-phase, a photoperiod-insensitive post-inductive phase under SD conditions.

## Results

### Flower Bud Initiation and Full Flower Opening

The average number of days to flowering was lower for control plants grown continuously under LD compared to those under SD (**Table 3**). In the photoperiod-sensitive accessions, [CDC Corinne (S1) and CDC Frontier (S2)], flowering time was delayed by 45 and 38 days, respectively, under SD compared to LD. Delay in flowering of the four accessions with intermediate response to photoperiod [ICC 15294 (I1), ICC 8621 (I2), ILC1867 (I3), and ICC 8855 (I4)] ranged from 17 to 42 days under SD compared to LD. Flowering in the day-neutral accessions, [ICCV 96029 (N1) and FLIP 98-142C (N2)], was delayed by 1 and 10 days, respectively, under SD as compared to LD.

**Table 3:** Average number of days from seeding to first flower bud initiation and the number of days to flower under long and SD photoperiod conditions over two time replicates of the experiment.

Accessions	Days from seeding to flower bud initiation		Days from seeding to first flowering	
	LD ± s.d.	SD ± s.d.	LD ± s.d.	SD ± s.d.
CDC Corinne (S1)	38 ± 1.0	39 ± 1.0	63 ± 2.5	101 ± 1.6
CDC Frontier (S2)	30 ± 1.0	38 ± 0.0	60 ± 3.3	105 ± 0.9
ICC 15294 (I1)	28 ± 1.0	31 ± 1.0	32 ± 0.5	74 ± 2.9
ICC 8621 (I2)	30 ± 0.0	30 ± 12	38 ± 1.3	55 ± 4.1
ILC 1687 (I3)	28 ± 1.0	31 ± 0.0	41 ± 1.3	66 ± 0.0
ICC 8855 (I4)	29 ± 0.0	30 ± 1.0	38 ± 1.3	68 ± 1.6
ICCV 96029 (N1)	19 ± 0.0	19 ± 0.0	28 ± 0.6	29 ± 0.0
FLIP 98-142C (N2)	22 ± 0.0	24 ± 0.0	30 ± 0.0	40 ± 0.8

### Slope Coefficients, *y*-Intercepts and Hinges

The slope coefficients, *y*-intercepts and the first and second hinges as determined by the simultaneous linear equations are listed in (**Table 4**). There were significant differences among the chickpea accessions for the second hinge, intercepts (a1, a22, and a3) and slope coefficients (b1, b2, and b22; *P* ≤ 0.0001). However, the difference among the accessions for the first hinge, the initial intercept (a2), and the slope coefficients (b3) of the simultaneous equations were not significant (**Table 5**).

**Table 4:** Means comparison of the hinge 1, a1, b1, a2, b2, hinge 2, and a22, b22, a3, and b3 for eight chickpea accessions evaluated in a reciprocal transfer experiment from LD (16 h light) and SD (10 h light) photoperiod conditions over two time replicates.

Accessions	Hinge 1	a1	a2	b1	b2	Hinge 2	a22	a3	b22	b3
CDC Corinne (S1)	20^a^	98^a^	123^a^	-0.3^a^	-1.6^bd^	42^a^	127^a^	60^ab^	-1.7^c^	0.0^a^
CDC Frontier (S2)	16^ab^	101^a^	120^a^	0.2^a^	-1.1^bbd^	47^a^	117^a^	75^a^	-1.1^bc^	-0.2^a^
ICC 15294 (I1)	20^a^	76^b^	115^ab^	-0.3^a^	-2.0^d^	45^a^	96^b^	34^c^	-1.4^c^	0.0^a^
ICC 8621 (I2)	9^ac^	59^d^	67^dc^	0.0^a^	-0.9^bbd^	26^bc^	76^c^	40^bc^	-1.4^c^	0.0^a^
ILC 1687 (I3)	10^abc^	68^c^	80^bc^	0.1^a^	-1.2^ddb^	36^ab^	82^bc^	36^bc^	-1.3^bc^	0.1^a^
ICC 8855 (I4)	3^bc^	34^f^	31^e^	0.0^a^	1.2^ab^	19^c^	11^f^	72^a^	3.6^a^	-0.3^a^
ICCV 96029 (N1)	0^c^	28^g^	28^e^	0.0^a^	0.0^ab^	1^d^	29^e^	27^c^	-3.0^d^	0.0^a^
FLIP 98-142C (N2)	9^abc^	40^e^	44^de^	-0.1^a^	-0.5^bc^	26^bc^	44^d^	32^c^	-0.5^bc^	-0.1^a^

*Values with the same letter in the same column are not significantly different; a1, a2, a22, and a3, stand for the y-intercepts (number of days to flowering); b1, b2, b3, b22, correspond to the slope coefficients for the accessions*.

**Table 5:** Analysis of variance for a1, b1, a2, b2, hinge 1, a22, b22, a3, b3, and hinge 2 of the eight chickpea accessions used in the photoperiod-sensitive and -insensitive phase determination using reciprocal transfers between SD (10 h light) and LD (16 h light) photoperiod conditions over two time replicates.

Characters	Source of variation			
	df	Sum of squares	Mean square	*F*-value
a1	7	10995	1571	278***
b1	7	22342	3192	13***
a2	7	0.2	0	1.3^ns^
b2	7	14	2	6.6**
Hinge 1	7	805	115	2.9^ns^
a22	7	30209	4316	100***
b22	7	5136	734	6.6**
a3	7	52	8	57***
b3	7	0.2	0	0.97^ns^
Hinge 2	7	3406	487	15***

a1, a2, a22, and a3 correspond to y-intercepts in relation to change in number of days to flowering under the respective photoperiods; b1, b2, b22, and b3, stand for slope coefficients in relation to the changes in time from seeding to first flowering against time from seeding to first transfer. *** and ** indicates significant difference at P ≤ 0.01 and 0.001, respectively and ns = not significant.

In our analysis, the first hinge corresponds to a beginning of change in time from seeding to first flowering against time from seeding to transfer. The photoperiod-sensitive accessions had the highest values of both the first and second hinge values. The intermediate accessions had intermediate values of both first and second hinges. The values of the first and second hinges for the day-neutral accession, ICCV 96029 (N1) were identified to be 0. The identified hinges facilitated determination of photoperiod-sensitive phase in chickpea accessions. The difference between hinge 1 and hinge 2 was considered as the photoperiod-sensitive phase. Accordingly, in CDC Frontier (S2), a photoperiod-sensitive accession, the first hinge and second hinge were 16 and 47 days, respectively. Based on the difference between the first and the second hinges, 31 days was considered as the length of the photoperiod-sensitive phase of this accession. Similarly, CDC Corinne (S1) and ICC 15294 (I1) each had the first hinge value of 20 days. The values of the second hinge for these two accessions were 42 and 45 days, respectively. The duration of photoperiod sensitivity of these accessions based on the difference between the second and first hinge were 22 and 25 days, respectively. For other intermediate accessions, the values of the first hinge were 9 to 10 days. The second hinge for these accessions was 19 days. Thus the duration of the photoperiod-sensitive phase ranged from 15 to 26 days.

### Linear Regression

The slope coefficient values of the accessions were negative for transfers from long to SD ranging from –0.40 to –1.00 (**Table 6**). On the other hand, the slope values of the accessions transferred from short to LD were positive ranging from 0.05 to 0.95. The slopes for ICCV 96029 (N1) transferred from LD to SD and from SD to LD were 0 and 0.05, respectively.

**Table 6:** Hinge regression for the eight chickpea accessions evaluated in the reciprocal transfer from LD to SD.

Experiment	Accessions	*y*-intercept (days)	slope coefficients (day/day)	*R*^2^ (%)	CV (%)
Days to flowering of the accessions transferred from long to SD	CDC Corinne (S1)	97	-0.55	0.9	9
	CDC Frontier (S2)	104	-0.65	0.94	5
	ICC 15294 (I1)	79	-0.8	0.91	11
	ICC 8621 (I2)	59	-0.45	0.81	9
	ILC1687 (I3)	69	-0.65	0.86	10
	ICC 8855 (I4)	34	-1	0.67	8
	ICCV 96029 (N1)	28	0	0.33	2
	FLIP98-142C (N2)	42	-0.4	0.88	5
Days to flowering of the accessions transferred from short to LD	CDC Corinne (S1)	53	0.6	0.92	6
	CDC Frontier (S2)	58	0.6	0.92	6
	ICC 15294 (I1)	31	0.7	0.92	8
	ICC 8621 (I2)	40	0.4	0.92	5
	ILC1687 (I3)	39	0.6	0.9	7
	ICC 8855 (I4)	73	0.95	0.85	11
	ICCV 96029 (N1)	28	0.05	0.64	2
	FLIP98-142C (N2)	29	0.3	0.77	5

*The y-intercept and slopes presented here are combined over two time replicates in growth chamber. CV, coefficient of variation*.

### Photoperiod-Sensitive and Photoperiod-Insensitive Phases in Chickpea Accessions

The reciprocal transfer model fitted the data with *R*^2^-values among the accessions ranging from 0.74 to 0.99 (**Table 7**). Three developmental phases were identified in all the accessions except ICCV 96029 (N1).

**Table 7:** Duration in days of each of the three developmental phases (*I*_1L_, *I*_1S_, *I*_2L_, *I*_2S,_ and *I*_3L_ and *I*_3S_) ± SE from seeding to first flower appearance in eight chickpea accessions under LD and SD conditions.

Accessions	I_1L_	I_1S_	I_2L_	I_2S_	I_3L_	I_3S_	f_L_	f_S_	R^2^
CDC Corinne (S1)	19 ± 3	17 ± 7	17 ± 6	49 ± 14	20 ± 5	29 ± 12	56	95	0.99
CDC Frontier (S2)	15 ± 7	20 ± 12	38 ± 9	77 ± 16	12 ± 6	4 ± 10	66	101	0.98
ICC 15294 (I1)	13 ± 9	18 ± 15	19 ± 11	43 ± 22	1.4 ± 7	15 ± 18	35	76	0.97
ICC 8621 (I2)	10 ± 1	13 ± 3	8 ± 2	25 ± 4	23 ± 1	23 ± 2	42	61	0.99
ILC 1687 (I3)	12 ± 10	12 ± 5	43 ± 14	16 ± 9	19 ± 10	17 ± 6	45	74	0.95
ICC 8855 (I4)	9 ± 5	14 ± 3	18 ± 6	32 ± 5	11 ± 4	19 ± 8	38	65	0.97
ICCV 96029 (N1)	22 ± 0.5	23 ± 0.3	0.1 ± 0.9	0	5 ± 0.9	5 ± 0	27	28	0.74
FLIP98-142C (N2)	18 ± 2	20 ± 3	7 ± 4	15 ± 5	5 ± 2	6 ± 3	30	41	0.98

*The values for the three developmental phases were derived from the experiment conducted in two time replicates and the mean values were used in the model to derive the values for each accession. I_1L_ = photoperiod-insensitive pre-inductive phase under LD, I_1S_ = photoperiod-insensitive pre-inductive phase under SD. I_2L_ = photoperiod-sensitive inductive phase under LD, I_2S_ = photoperiod-sensitive inductive phase under SD. I_3L_ = photoperiod-insensitive post-inductive phase under LD, I_3S_ = photoperiod-insensitive post-inductive phase under SD. f_L_ = days from seeding to flowering under LD, f_S_ = days from seedling seeding to flowering under SD. R^2^ = the amount of variation accounted for by the model.*

#### Photoperiod-Insensitive Pre-inductive Phase

In the photoperiod-sensitive accessions, a photoperiod-insensitive pre-inductive phase of 15–19 days was observed under LD, and 17–20 days under SD. These values ranged from 9 to 13 days in the intermediate accessions under LD and from 13 to 18 days under SD. In ICCV 96029 (N1), the values of the photoperiod-insensitive pre-inductive phase were 22 and 23 days under long and SD, respectively. This value ranged between 18 and 20 days under long and SD, respectively, in FLIP 98-142C (N2).

#### Photoperiod-Sensitive Inductive Phase

The two photoperiod-sensitive accessions, [CDC Corinne (S1) and S2 (CDC Frontier (S2)] had higher values for the photoperiod-sensitive inductive phase under SD compared to LD. In these accessions, the photoperiod-sensitive inductive phase under LD ranged from 17 to 38 days, and were 49 to 77 under SD. In the accessions with intermediate ICC 15294 (I1), ICC8621 (I2), ILC1867 (I3), and ICC 8855 (I4) response to photoperiod the photoperiod-sensitive inductive phase under LD ranged from 12 to 19 days, and was 25 to 43 under SD.

For ICCV 96029 (N1), the values of the photoperiod-sensitive inductive phase under LD and SD were 0.1 and 0.0, respectively. In FLIP98-142C (N2), another photoperiod-insensitive accession, the range of the photoperiod-sensitive inductive phase under LD and SD was 7 and 15 days, respectively. In the highly photoperiod-sensitive accessions, the photoperiod-insensitive post-inductive phase ranged between 13 and 20 days under LD, and between 4 and 29 days under SD, respectively. In the intermediate accessions, this phase ranged between 5 and 20 days under LD, and between 15 and 23 days under SD. The photoperiod-insensitive accessions had a similar range for the photoperiod-insensitive post-inductive phase of 5–6 days in long as well as in SD.

#### Photoperiod-Insensitive Post-inductive Phase

In the highly photoperiod-sensitive accessions, the photoperiod-insensitive post-inductive phases were between 13 and 20 days under LD and between 4 and 29 days under SD, respectively. In the intermediate accessions, the values of this phase ranges between 5 and 20 days under LD and between 15 and 23 days under SD. The photoperiod-insensitive accessions had similar range of photoperiod-insensitive post-inductive phases of 5 to 6 days in long as well as SD.

## Discussion

When control plants in the respective chambers were compared, the plants under LD flowered earlier than those under SD. Early transfer of plants from either long to SD chambers or vice versa had no effect on the flowering response of the plants. Differences in the number of days to flower between SD and LD control plants were wider compared to the number of days to flower bud initiation. This indicated that day to full flower opening was delayed by short photoperiods after flower bud initiation. Wallace et al. (1993) reported that time to initiation of flower buds could not be used to differentiate the insensitive and sensitive genotypes in soybean.

The slope coefficients of the flowering responses under non-optimal photoperiods provided an estimate of photoperiod sensitivity (Major, 1980). In our study, the absolute values of the slopes for the photoperiod-sensitive and intermediates were greater than the day-neutral ones. (ICCV 96029 (N1) specifically had slopes of 0 and 0.05 for transfers from LD to SD and SD to LD, respectively. Both values were not significantly different from 0, supporting the previous report (Daba et al., 2015) that this accession is day-neutral under a mean temperature of 19°C combined with either 10 or 16 h photoperiod.

The hinge regression function technique was exploited to identify photoperiod-sensitive and -insensitive phases in the chickpea accessions. The hinge technique was very efficient in differentiating between photoperiod-sensitive and -insensitive phases in the photoperiod-sensitive and intermediate accessions. The actual data used to determine the photoperiod sensitivity in most plants seldom resemble the idealized schematic diagram similar to the response of FLIP 98-142C (N2) and CDC Frontier (S2) in our research. Hinge regression functions are applied most importantly in multivariate regression and classifications. In our study, the advantage of the hinge regression function was evident in the day-neutral accession ICC V96029 (N1) for which the first and second hinges were 0 and 1, respectively, indicating the absence of a significant change in the flowering response, and confirming that ICCV 96029 is day-neutral under our experimental conditions.

Chickpea, therefore, has three flowering induction phases: a photoperiod-insensitive pre-inductive phase, a photoperiod-sensitive inductive phase, and photoperiod-insensitive post-inductive phase. An inverse relationship between photoperiod sensitivity phase and photoperiod was identified, i.e., a longer photoperiod-sensitive phase was observed under SD, and a shorter photoperiod-sensitive phase was observed under LD. Variability in the length of the photoperiod-insensitive pre-inductive phase was observed among the photoperiod-sensitive, intermediate, and day-neutral chickpea accessions. A shorter duration of photoperiod-insensitive pre-inductive phase was detected compared to the photoperiod-sensitive phase in intermediate accessions. During the photoperiod-insensitive pre-inductive phase, plants were not responsive to changes in the photoperiod. In many crops, a minimum vegetative period, known as the basic vegetative phase, is required during which there is no response to photoperiod (Vergara and Chang, 1985).

The two high yielding accessions developed and released by the Crop Development Centre, University of Saskatchewan [CDC Corinne (S1) and CDC Frontier (S2)] (Warkentin et al., 2005; Tar’an et al., 2009) had the longest time to flowering, as well as longer duration of photoperiod sensitivity phases under LD and SD. Daba et al. (2015, 2016) reported that ICCV 96029 (N1) and FLIP98-142C (N2) flowered the earliest; ICC 15294 (I1), ICC 8621 (I2), ILC 1867 (I3), and ICC 8855 (I4) flowered intermediate and CDC Frontier (S2) and CDC Corinne (S1) flowered the latest under a combination of temperature and photoperiod in the growth chamber conditions.

Efforts to develop early flowering cultivars adapted to the short growing season of western Canada could exploit ICCV 96029 (N1) and FLIP 98-142C (N2), which have a minimal photoperiod-sensitive phase. This strategy was also recommended by Kumar and Abbo (2001). Photoperiod-insensitivity contributed a significant share for chickpea adaptation to low latitude during early domestication (Siddique et al., 2003; Rubio et al., 2004). In chickpea, the number of biological days from emergence to flowering should match the latitude locations based on photoperiod sensitivity (Vadez et al., 2013). Early flowering and maturity in photoperiod-insensitive genotypes in bean has helped to attain higher harvest index compared to the photoperiod-sensitive genotypes (Yourstone et al., 1993).

## Conclusion

The phenology of chickpea accessions from emergence to first flowering can be divided into three phases: (1) a photoperiod-insensitive pre-inductive phase, (2) a photoperiod-sensitive inductive phase, and (3) a photoperiod-insensitive post-inductive phase. The duration of the photoperiod-insensitive pre-inductive phase was shorter than that of the photoperiod-sensitive inductive phase in chickpea. Photoperiod sensitivity commenced on different days after emergence in different accessions. The photoperiod-sensitive inductive phase extended beyond flowering bud initiation and full flower opening to the stage of full flower development. Flower bud initiation and full flower opening appeared to be sensitive to photoperiod at different times after emergence for different chickpea accessions. Time to flower bud initiation as well as time to full flower opening differentiated photoperiod-insensitive and photoperiod-sensitive accessions. In the cool short seasons of Western Canada, chickpea accessions with a shorter duration both the pre-inductive photoperiod-insensitive and photoperiod-sensitive inductive phases are desirable for adaptation. The day-neutral accessions such as ICCV 96029 and FLIP98-142C are used for developing cultivars fit to the tropics, subtropics, and the Mediterranean regions characterized by short growing seasons delimited by increasing temperatures and reduced soil moisture where short crop duration is desired.

## Author Contributions

KD conducted the experiments, analyzed, and summarized the results. KD, TW, RB, CT, and BT wrote and finalized the manuscript; BT and TW conceived and directed the project.

## Conflict of Interest Statement

The authors declare that the research was conducted in the absence of any commercial or financial relationships that could be construed as a potential conflict of interest.

## Abbreviations

LD: long day
PS: photoperiod sensitivity
SD: short day
